# Supplementary material for: Electric field dynamics in the brain during multi-electrode transcranial electric stimulation
Source: Nat Commun. 2019 Jun 12;10:2573. doi: 10.1038/s41467-019-10581-7 (PMC6561925; doi:10.1038/s41467-019-10581-7)
Supplement: Supplementary file 1 — Supplementary Information [file 41467_2019_10581_MOESM1_ESM.pdf]

Supplementary Discussion

Here, we investigate the electric fields during three-electrode transcranial alternating current stimulation (TACS). The electrodes were placed on the scalp over the middle forehead (first stimulation electrode), the left occipital area (second stimulation electrode), and the left temporal area (return electrode). While the exact amplitude A and phases φ_1 and φ_2 of the currents that are passed via two main stimulation electrodes are precisely controlled, the amplitude and phase of the return current are not explicit stated. One can derive the return current analytically as follows:

$$I_1 = A\sin(\omega t) \quad (1)$$

$$I_2 = A\sin(\omega t + \varphi_{\mathrm{S}}) \quad (2)$$

$$I_{\mathrm{R}} = -(I_1 + I_2) = -A(\sin(\omega t) + \sin(\omega t + \varphi_{\mathrm{S}})) \quad (3)$$

$$I_{\mathrm{R}} = -A\left(2\sin\left(\frac{\omega t + (\omega t + \varphi_{\mathrm{S}})}{2}\right)\cos\left(\frac{\omega t - (\omega t + \varphi_{\mathrm{S}})}{2}\right)\right) \quad (4)$$

$$I_{\mathrm{R}} = -2A\sin\left(\omega t + \frac{\varphi_{\mathrm{S}}}{2}\right)\cos\left(\frac{\varphi_{\mathrm{S}}}{2}\right) \quad (5)$$

Where, I_1 – the current of electrode 1 in Amperes, I_2 – the current of electrode 2, I_{R} – the return current, t – the time in seconds, ω – the angular frequency in radians per second ($\omega = 2\pi f$, where f – frequency in Hz), φ_{S} – the phase difference between the second (φ_2) and first (φ_1) stimulation electrode in radians.

This equation provides a closed form solution for the return current. Amplitude and phase of the return current depend on the phase difference of the stimulation electrodes. For a phase difference of 0 degrees return currents will be maximal, while for 180 degrees return currents are zero. The 180 degrees case corresponds to a classical two electrode TACS configuration. Current profiles for the return current for selected phase conditions are shown in Supplementary Figure 1.

For any given phases φ_1 and φ_2 of the input currents applied through the two stimulation electrodes with a common reference, we can determine the resulting electric field in the brain based on the superposition principle. For this, we transform the electric fields $\mathbf{E}_1$ and $\mathbf{E}_2$ resulting from stimulation electrode 1 with phase φ_1 and stimulation electrode 2 with phase φ_2 (with the common reference electrode) into their phasor form $\mathbf{P}_1$ and $\mathbf{P}_2$. Below we derive the equations for the x-component of the electric field (y and z components can be derived in the same way):

$$P_1^{\mathrm{x}} = E_1^{\mathrm{x}}\, cos\,\varphi_1 + \mathrm{j}(E_1^{\mathrm{x}}\, sin\,\varphi_1) \quad (6)$$

$$P_2^{\mathrm{x}} = E_2^{\mathrm{x}}\, cos\,\varphi_2 + \mathrm{j}(E_2^{\mathrm{x}}\, sin\,\varphi_2) \quad (7)$$

To calculate the resulting electric field arising from three-electrode stimulation, we can exploit the superposition principle:

$$P_3^{\mathrm{x}} = (E_1^{\mathrm{x}} \cos\varphi_1 + E_2^{\mathrm{x}} \cos\varphi_2) + \mathrm{j}(E_1^{\mathrm{x}} \sin\varphi_1 + E_2^{\mathrm{x}} \sin\varphi_2) \quad (8)$$

From the resulting phasor $\mathbf{P}_3$ we can estimate the phase $\varphi_{\mathrm{E}}^{\mathrm{x}}$ and amplitude $|E^{\mathrm{x}}|$ of the resulting electric field as:

$$\varphi_{\mathrm{E}}^{\mathrm{x}} = \tan^{-1} \frac{(E_1^{\mathrm{x}} \sin\varphi_1 + E_2^{\mathrm{x}} \sin\varphi_2)}{(E_1^{\mathrm{x}} \cos\varphi_1 + E_2^{\mathrm{x}} \cos\varphi_2)} \quad (9)$$

$$|E^{\mathrm{x}}| = \sqrt{(E_1^{\mathrm{x}} \cos\varphi_1 + E_2^{\mathrm{x}} \cos\varphi_2)^2 + (E_1^{\mathrm{x}} \sin\varphi_1 + E_2^{\mathrm{x}} \sin\varphi_2)^2} \quad (10)$$

Thus, phasor analysis provides a way for the precise characterization of phase relationships of the electric field during multi-electrode TACS with any arbitrary configuration of input currents. From Supplementary Equation 9 it follows that the resulting phase $\varphi_{\mathrm{E}}^{\mathrm{x}}$ will vary spatially (except when φ_1, φ_2 = 0 or 180 degrees). The resulting phase angle will depend on the ratio of E_1^{x} to E_2^{x}, with the dominant component determining its value (see Supplementary Figure 2). As the ratio of E_1^{x} and E_2^{x} will change with the location in the brain since these electric fields arise from two different subsets of electrodes, the resulting phase angle $\varphi_{\mathrm{E}}^{\mathrm{x}}$ will vary spatially (see Supplementary Figure 2). The experimentally measured electric field E_{meas} captures the component of the TACS electric field $\mathbf{E}$ along the direction $\mathbf{d}$ of the implanted electrode array:

$$E_{\mathrm{meas}} = \mathbf{d} \cdot \mathbf{E} \quad (11)$$

Considerations, as conducted here for the x-component, can be performed equivalently for y and z components or any arbitrary direction such as the direction of the implanted electrode array. The phasor formulation thus facilitates efforts to simulate multielectrode TACS electric fields using the finite element method which can be compared to the measured electric field (see example in Supplementary Figure 11).

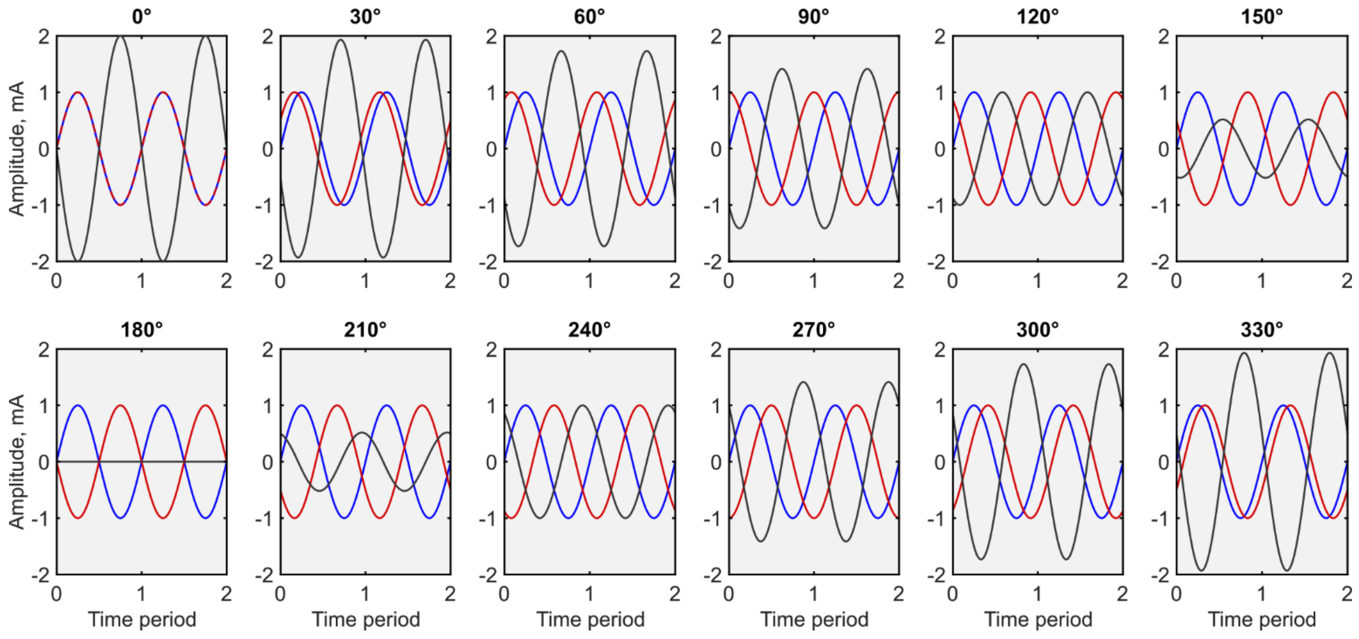

Supplementary Figure 1. Electric currents that are passed through the first stimulation (blue), second stimulation (red), and return (gray) electrodes depending on the phase configuration. The configuration varies in the phase difference between the currents that pass via two stimulation electrodes. In this illustration, the peak-to-zero intensities of stimulation currents are constant and equal to 1 mA.

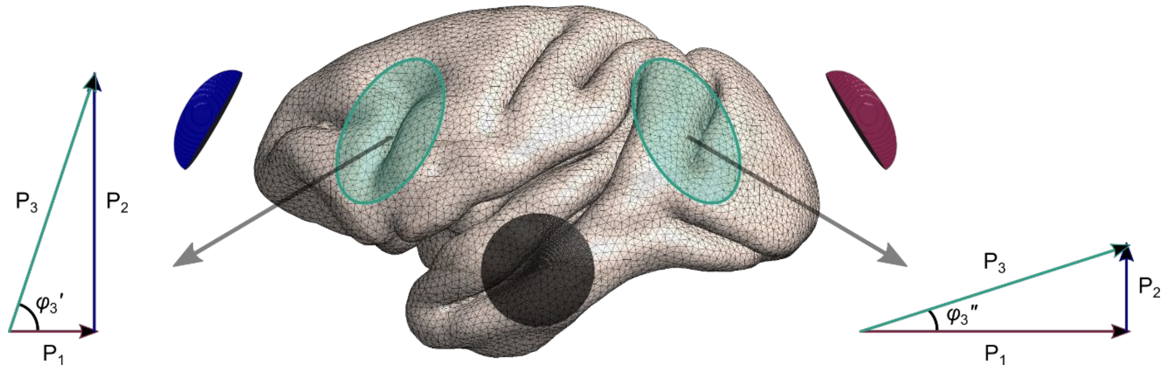

Supplementary Figure 2. Illustration of the phasor addition principle. A phasor $\mathbf{P}_1$ with φ_1 = 0 degrees and a phasor $\mathbf{P}_2$ with φ_2 = 90 degrees are added at different brain locations. The resulting phasor $\mathbf{P}_3$ will exhibit a phase angle φ_3 according to Supplementary Equation 9 which will vary across different brain regions.

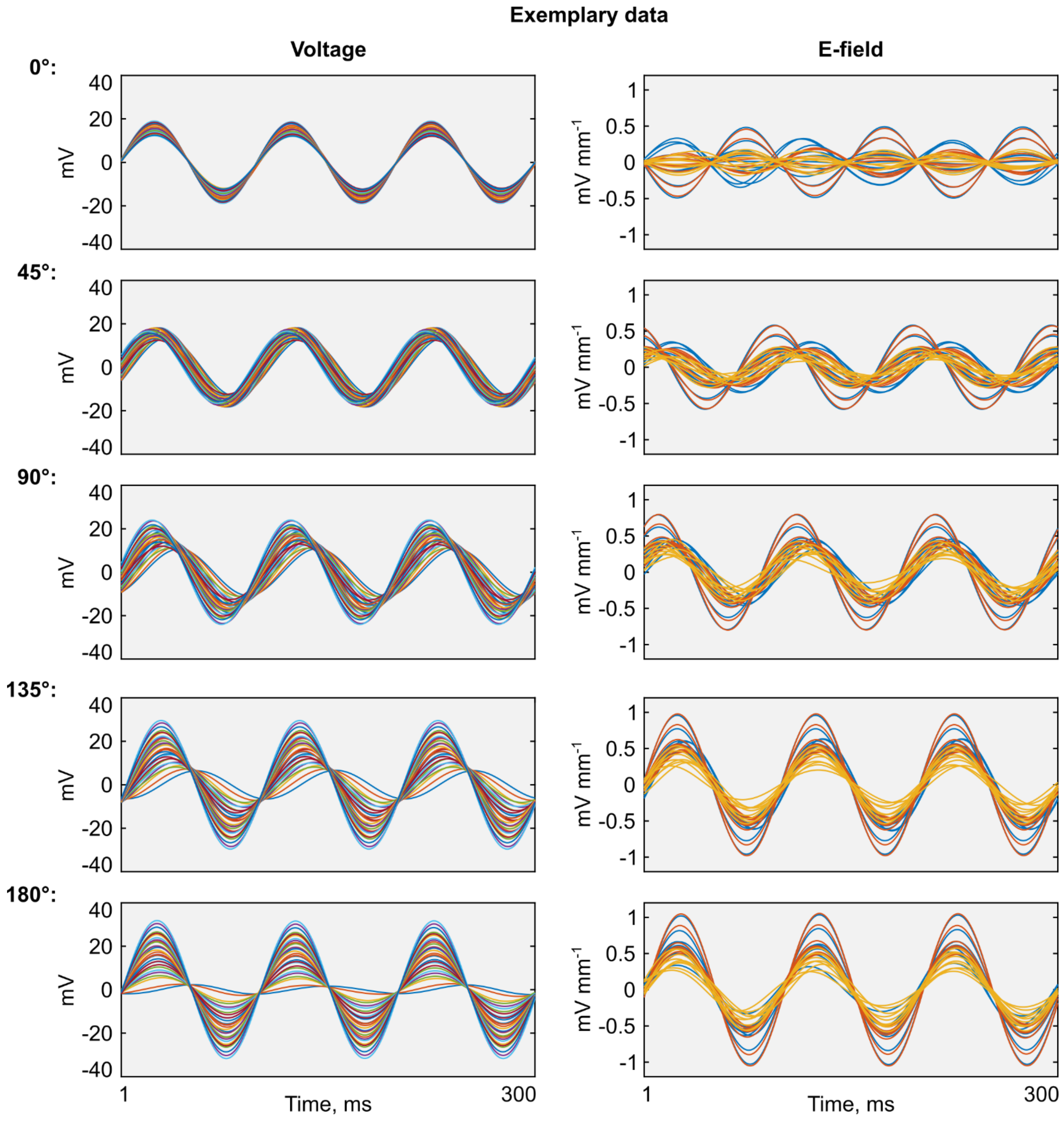

Supplementary Figure 3. Examples of preprocessed recordings from the implanted electrodes during TES for five stimulation conditions (from top to bottom: 0°, 45°, 90°, 135° and 180° stimulation phase differences between the anterior and posterior stimulation electrodes). Here and everywhere, the units of the raw data are scaled to mV (for the voltages) or mV mm^{-1} (for the electric field) per 1 mA peak-to-zero of transcranially applied electric current.

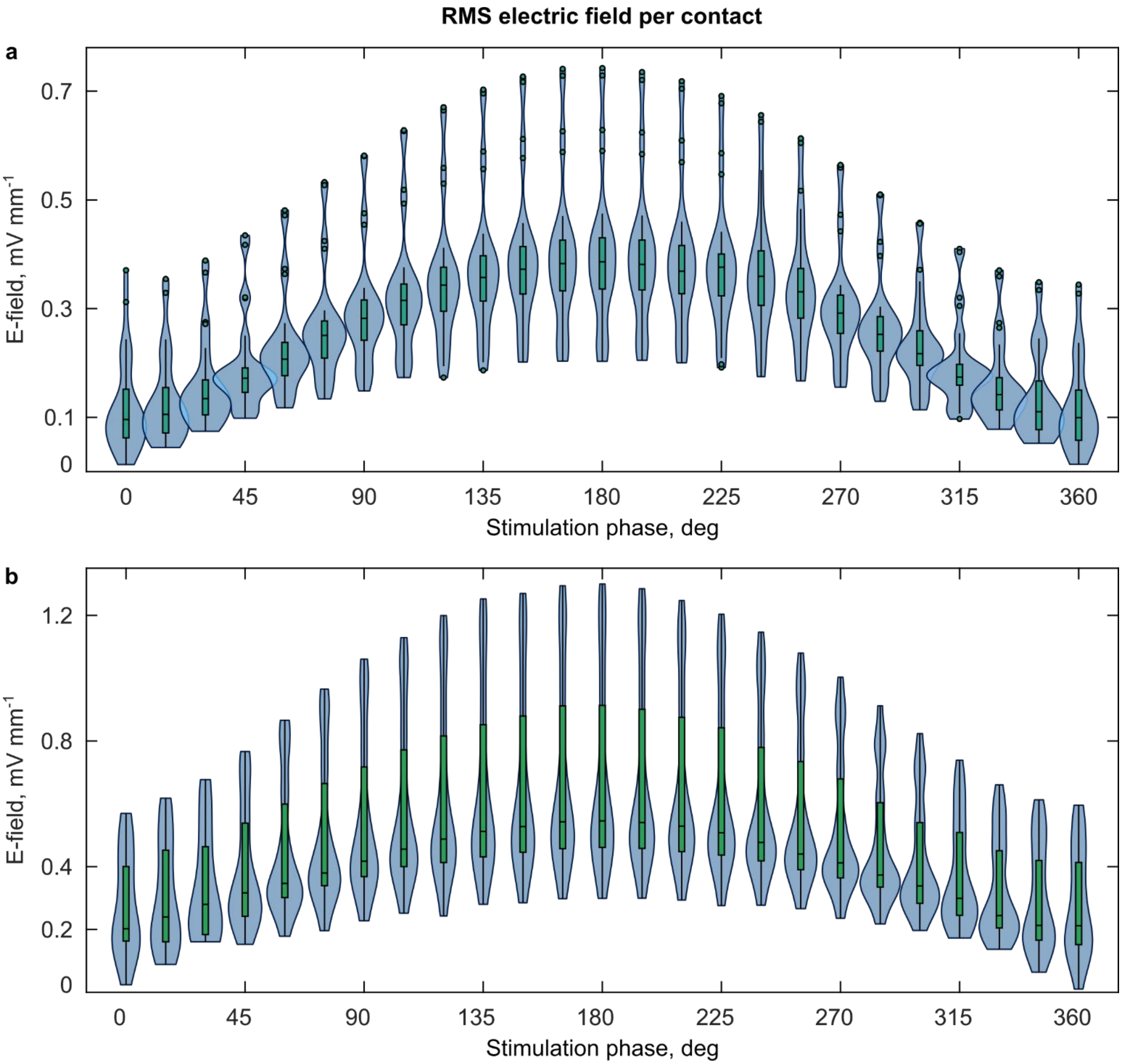

Supplementary Figure 4. Violin plots of the RMS magnitude of the electric field in the brain. The x-axis indicates the stimulation condition (i.e., the phase difference between the anterior and posterior stimulation electrodes). **a** The upper plot corresponds to subject 1, and **b** the lower plot to subject 2. The kernel density outlines are normalized to have the same area. The units are scaled to mV mm^{-1} per 1 mA peak-to-zero of applied current. This figure complements Figure 3 in the main paper.

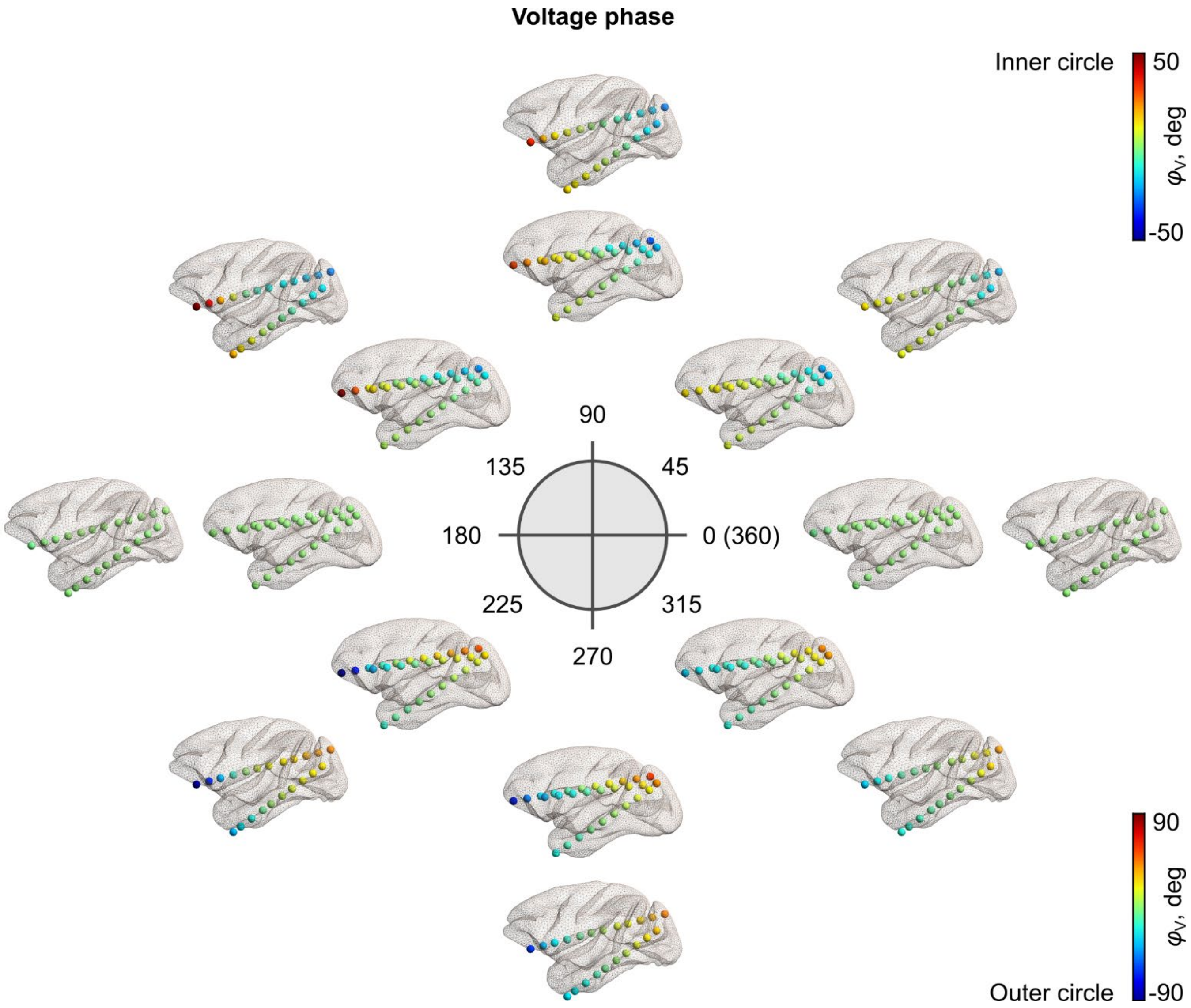

Supplementary Figure 5. Voltage phases in the brain during TES. The phase difference between the anterior and posterior stimulation electrodes is indicated in the middle plot. The inner circle of plot corresponds to subject 1, and the outer circle to subject 2. This figure complements Figure 4 in the main paper.

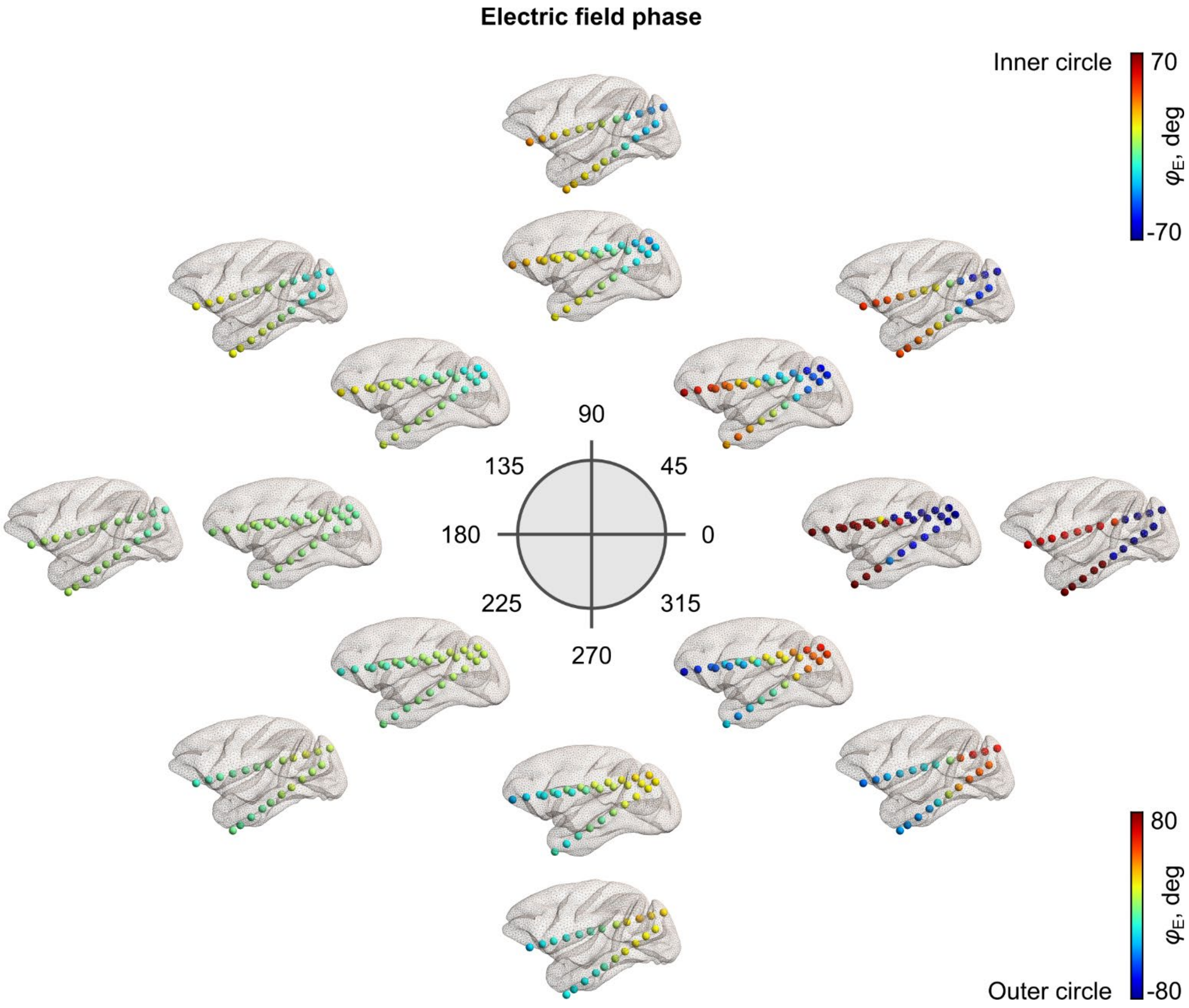

Supplementary Figure 6. Electric field phases in the brain during TES. The phase difference between the anterior and posterior stimulation electrodes is indicated in the middle plot. The inner circle of plot corresponds to subject 1, and the outer circle to subject 2. This figure complements Figure 4 in the main paper.

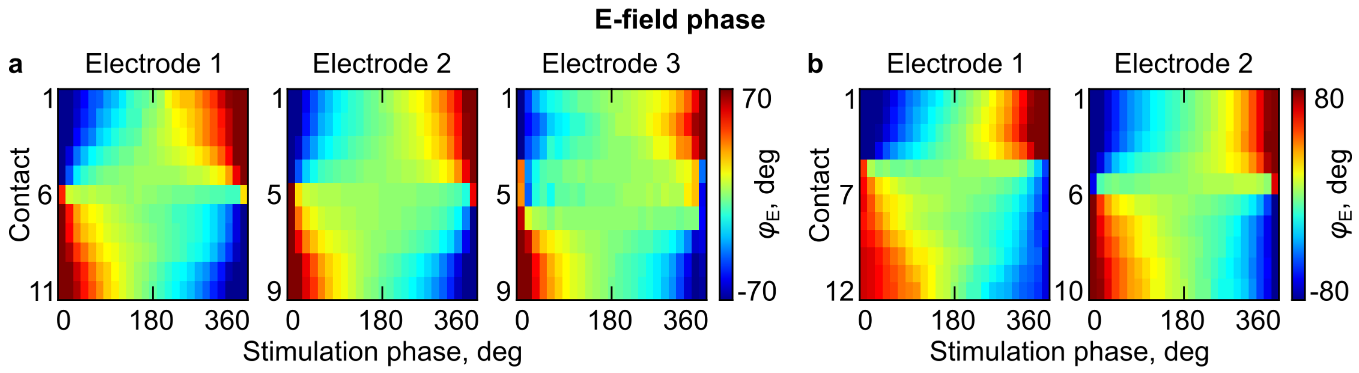

Supplementary Figure 7. Local electric field phase in the brain during TES for a given stimulation condition in subject 1 (**a**) and 2 (**b**). The first contact for each electrode corresponds to the most posterior location, and the last contact – to the most anterior location, with 5 mm spacing between adjacent contacts. This figure complements Figure 4 in the main paper.

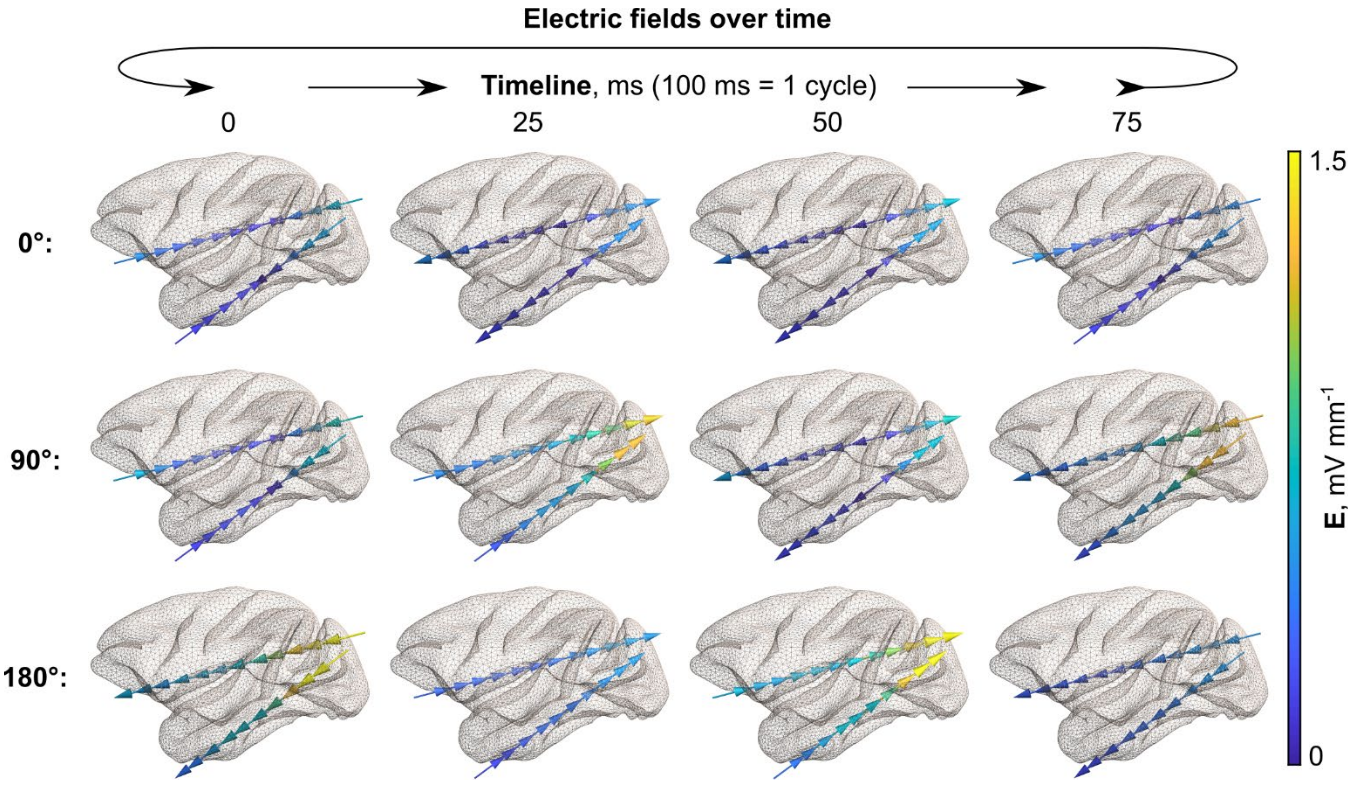

Supplementary Figure 8. Electric fields in the brain over time during TES for a given stimulation condition for subject 2. The panel depicts the main conditions (0°, 90°, and 180° stimulation phase differences) for subject 2. Arrows indicate the electric field direction, and the color encodes the electric field magnitude in mV mm^{-1}. This figure complements Figure 5 in the main paper.

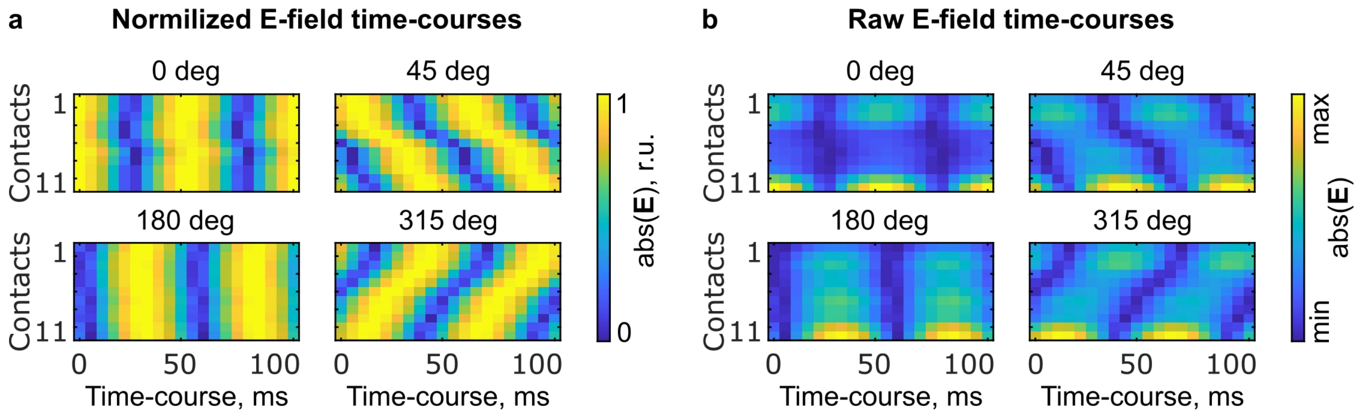

Supplementary Figure 9. a Absolute normalized and **b** non-normalized electric field time-courses for subject 1 and electrode 1. The first contact in the electrode corresponds to the most anterior location, and the last contact – to the most posterior location. While for 0° and 180° the maxima across contacts occur at the same time point, they occur at different time points for the 45° condition (= traveling wave). Other electrodes demonstrate a similar pattern. The panel corresponds to Figure 6 in the main paper. Also see Supplementary Movies 4-7 for a 3d animation.

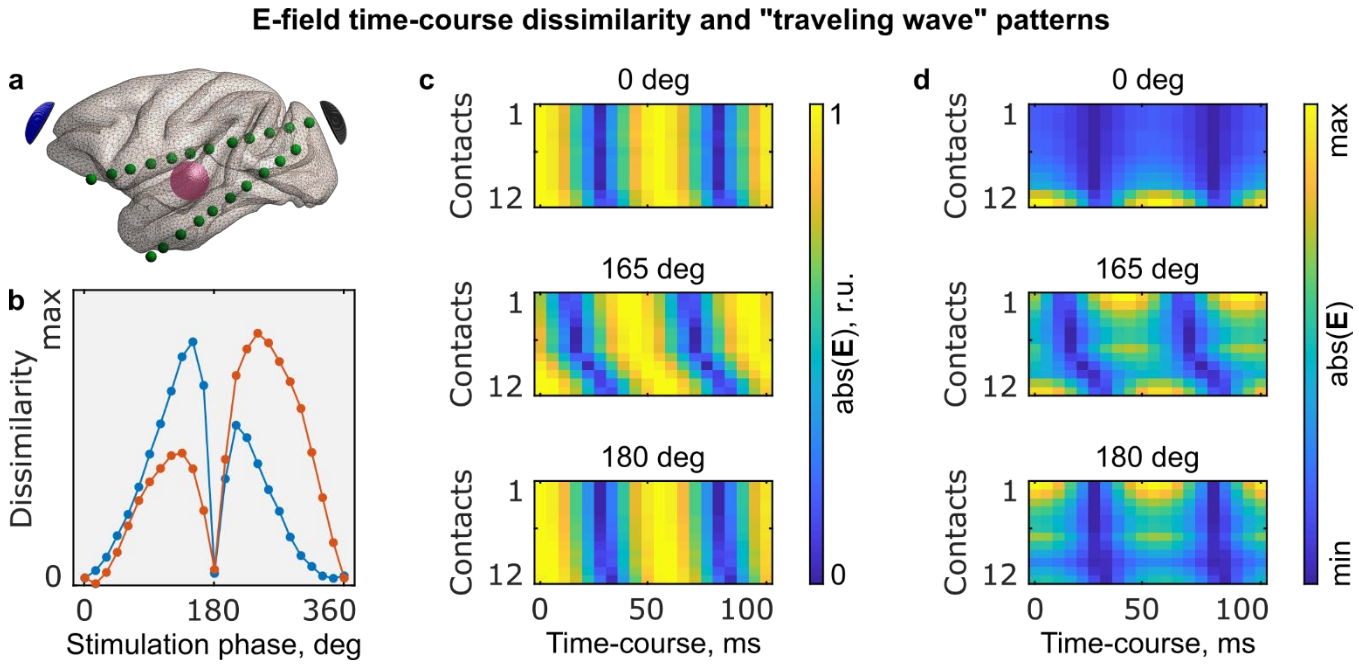

Supplementary Figure 10. a Control stimulation condition. The stimulation electrode montage and, therefore, electric field in the brain are rotated by 90 degrees relative to the main experiment: one active electrode is located on the forehead, another – on the left temple, and the return electrode – on the back of the head. **b** Dissimilarity between the electric field time-courses at different contacts of the first (blue) and second (red) electrodes. **c** Absolute normalized and **d** non-normalized electric field time-courses for electrode 1. The first contact in the electrode corresponds to the most anterior location, and the last contact – to the most posterior location. While for 0° and 180° the maxima across contacts occur at the same time point, they occur at different time points for the 165° condition (= traveling wave). The second electrode demonstrates a similar pattern. The panel corresponds to Figure 6 in the main paper.

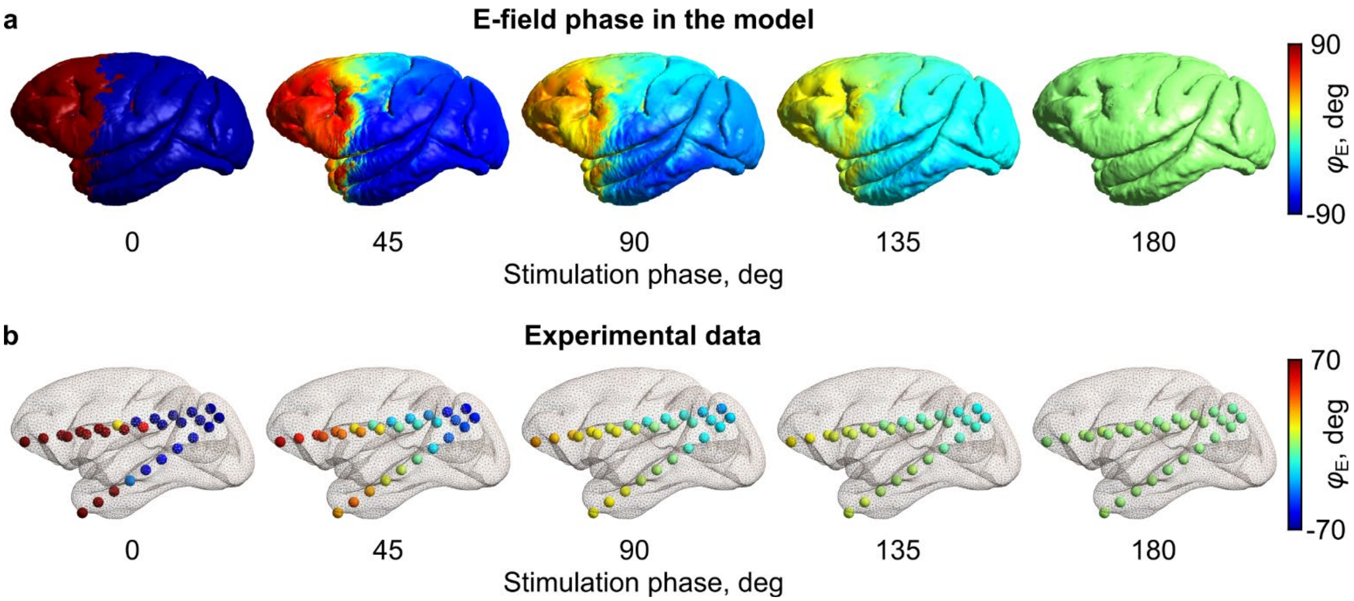

Supplementary Figure 11. Phasor analysis of the electric fields in the computer simulation. **a** The analytical phase on the brain surface in the model. **b** The experimental recordings from the invasive electrodes in subject 1. This figure complements Figure 7 in the main paper.

Supplementary Table 1. Locations of the recording contacts in the brain.

Subject	Electrode	Anatomical locations (from more posterior to more anterior structures)
1	1	Area V1, Area V2, Area V3, Area V4, Ventral intraparietal area, Corpus callosum, Caudate, Brodmann Area 24, Brodmann Area 32, Brodmann Area 25
	2	Area V1, Area V2, Area V3, Area V4, Ventral intraparietal area, Medial superior temporal area, Pulvinar, Frontal eye field
	3	Area V1, Area V2, Area V1 peripheral, Area V2 peripheral, Hippocampus, Parahippocampal area
2	1	Area V1, Areas V2/V4, Area MT, Ventral intraparietal area, White matter, Pulvinar/Caudate, Caudate/Corpus callosum, Caudate, Brodmann Area 25
	2	Area V1, Area V2, Area V3, Area V1 peripheral, Area V2 peripheral, Hippocampus, Area V4, Parahippocampal areas
