# Supplementary material for: Electric field dynamics in the brain during multi-electrode transcranial electric stimulation
Source: Nat Commun. 2019 Jun 12;10:2573. doi: 10.1038/s41467-019-10581-7 (PMC6561925; doi:10.1038/s41467-019-10581-7)
Supplement: Supplementary file 10 — Reporting Summary [file 41467_2019_10581_MOESM10_ESM.pdf]

nature research

Corresponding author(s): Alexander Opitz

Last updated by author(s): May 7, 2019

Reporting Summary

Nature Research wishes to improve the reproducibility of the work that we publish. This form provides structure for consistency and transparency in reporting. For further information on Nature Research policies, see Authors & Referees and the Editorial Policy Checklist.

Statistics

For all statistical analyses, confirm that the following items are present in the figure legend, table legend, main text, or Methods section.

n/a	Confirmed	
☐	☒	The exact sample size (*n*) for each experimental group/condition, given as a discrete number and unit of measurement
☐	☒	A statement on whether measurements were taken from distinct samples or whether the same sample was measured repeatedly
☐	☒	The statistical test(s) used AND whether they are one- or two-sided<br>*Only common tests should be described solely by name; describe more complex techniques in the Methods section.*
☐	☒	A description of all covariates tested
☐	☒	A description of any assumptions or corrections, such as tests of normality and adjustment for multiple comparisons
☐	☒	A full description of the statistical parameters including central tendency (e.g. means) or other basic estimates (e.g. regression coefficient) AND variation (e.g. standard deviation) or associated estimates of uncertainty (e.g. confidence intervals)
☐	☒	For null hypothesis testing, the test statistic (e.g. *F*, *t*, *r*) with confidence intervals, effect sizes, degrees of freedom and *P* value noted<br>*Give P values as exact values whenever suitable.*
☒	☐	For Bayesian analysis, information on the choice of priors and Markov chain Monte Carlo settings
☒	☐	For hierarchical and complex designs, identification of the appropriate level for tests and full reporting of outcomes
☒	☐	Estimates of effect sizes (e.g. Cohen's *d*, Pearson's *r*), indicating how they were calculated

Our web collection on statistics for biologists contains articles on many of the points above.

Software and code

Policy information about availability of computer code

Data collection	Data recording was perfromed using the software form the amplifier's manufacture: BrainAmp MR plus amplifier (Brain Products) and Cortech NeurOne Tesla amplifier (Cortech Solution).
Data analysis	Data were analyzed with MATLAB 2017b (MathWorks) using the Fieldtrip toolbox (RRID:SCR_004849) and Statistics toolbox (MathWorks).

For manuscripts utilizing custom algorithms or software that are central to the research but not yet described in published literature, software must be made available to editors/reviewers. We strongly encourage code deposition in a community repository (e.g. GitHub). See the Nature Research guidelines for submitting code & software for further information.

Data

Policy information about availability of data

All manuscripts must include a data availability statement. This statement should provide the following information, where applicable:

- Accession codes, unique identifiers, or web links for publicly available datasets
- A list of figures that have associated raw data
- A description of any restrictions on data availability

The data that support the findings of this study and the code are available from the corresponding author upon reasonable request.

Field-specific reporting

Please select the one below that is the best fit for your research. If you are not sure, read the appropriate sections before making your selection.

☒ Life sciences ☐ Behavioural & social sciences ☐ Ecological, evolutionary & environmental sciences

October 2018

Life sciences study design

All studies must disclose on these points even when the disclosure is negative.

Sample size	Experiments were conducted in two non-human primates. The sample size is based on the literature.
Data exclusions	For the subject 1, we excluded three contacts and interpolated them from neighboring sites due to excessive noise at these contacts. We further excluded the most posterior contact for each electrode because they were outside the grey matter. For subject 2, two contacts were excluded and interpolated from the neighboring sites. One electrode was entirely removed from the analysis due to low recording quality.
Replication	All measurements were repeated in two non-human primates from two taxonomic families with substantially different head anatomy. Both experiments yielded highly comparable results.
Randomization	Not applicable, because there were no experimental groups in the study.
Blinding	Not applicable to the study, because the research was performed on the anesthetized animals.

Reporting for specific materials, systems and methods

We require information from authors about some types of materials, experimental systems and methods used in many studies. Here, indicate whether each material, system or method listed is relevant to your study. If you are not sure if a list item applies to your research, read the appropriate section before selecting a response.

Materials & experimental systems

n/a	Involved in the study	
☒	☐	Antibodies
☒	☐	Eukaryotic cell lines
☒	☐	Palaeontology
☐	☒	Animals and other organisms
☒	☐	Human research participants
☒	☐	Clinical data

Methods

n/a	Involved in the study	
☒	☐	ChIP-seq
☒	☐	Flow cytometry
☒	☐	MRI-based neuroimaging

Animals and other organisms

Policy information about studies involving animals; ARRIVE guidelines recommended for reporting animal research

Laboratory animals	Experiments were conducted in two non-human primates. Subject 1 is a capuchin monkey (Cebus apella, 11 y.o., female, 2.9 kg) and subject 2 is a rhesus monkey (Macaca mulatta, 6 y.o., female, 4.8 kg).
Wild animals	The study did not involve wild animals.
Field-collected samples	The study did not involve samples collected from the field.
Ethics oversight	All procedures were approved by the Institutional Animal Care and Use Committee of the Nathan Kline Institute for Psychiatric Research (USA).

Note that full information on the approval of the study protocol must also be provided in the manuscript.
